# Changes in Smoking Behavior over Family Transitions: Evidence for Anticipation and Adaptation Effects

**DOI:** 10.3390/ijerph14060610

**Published:** 2017-06-07

**Authors:** Damien Bricard, Stéphane Legleye, Myriam Khlat

**Affiliations:** 1 Institut de Recherche et Documentation en Économie de la Santé (Irdes), 117 bis rue Manin, 75019 Paris, France; 2 Institut National d’Études Démographiques (Ined), 133 boulevard Davout, 75020 Paris, France; khlat@ined.fr; 3 Institut National de la Statistique et des Études Économiques (Insee), 6 Rue Legrand, 92240 Malakoff, France; stephane.legleye@insee.fr; 4 Centre de Recherche en Épidémiologie et Santé des Populations (CESP), Faculté de Médecine—Université Paris-Sud, Faculté de Médecine—UVSQ, INSERM, Université Paris-Saclay, 94805 Villejuif, France

**Keywords:** smoking, family life transitions, life cycle, longitudinal analysis, anticipation effects, adaptation effects

## Abstract

The study of changes in smoking behaviors over the life course is a promising line of research. This paper aims to analyze the temporal relation between family transitions (partnership formation, first childbirth, separation) and changes in smoking initiation and cessation. We propose a discrete-time logistic model to explore the timing of changes in terms of leads and lags effects up to three years around the event in order to measure both anticipation and adaptation mechanisms. Retrospective biographical data from the Santé et Itinéraires Professionnels (SIP) survey conducted in France in 2006 are used. Partnership formation was followed for both genders by a fall in smoking initiation and an immediate rise in smoking cessation. Childbirth was associated with increased smoking cessation immediately around childbirth, and additionally, females showed an anticipatory increase in smoking cessation up to two years before childbirth. Couple separation was accompanied by an anticipatory increase in smoking initiation for females up to two years prior to the separation, but this effect only occurred in males during separation. Our findings highlight opportunities for more targeted interventions over the life course to reduce smoking, and therefore have relevance for general practitioners and public policy elaboration.

## 1. Introduction

Over the past decades, the dissemination of information on the harmful effects of tobacco and the implementation of public policies have contributed to a gradual and continuous decrease in smoking prevalence. In fact, this decline in smoking was largely attributed to a decrease in prevalence in the most privileged groups and with different timing in men and women [1,2,3]. These evolutions have led to a sharp increase in social inequalities in smoking and a convergence of male and female prevalence.

At the present time, differences between men and women remain in the age pattern of smoking for the most recent cohorts: in the most educated group, women smoke regularly before the age of 25 more frequently than men, and do so less frequently after this age. These differences can probably be explained by a greater cessation of women at the approach of the their first pregnancy, especially among the most educated, and could also reflect the specificities of smoking patterns by gender and social group [4,5,6]. Exploring the dynamics of changes in smoking behaviors over the life course appears to be a promising line of research to understand the persistence as well as the evolution of smoking disparities over cohorts and over ages.

The period of the transition into adulthood is a period of life associated with the transitions to adult work and family roles and the related changes in health behaviors [7,8], which could explain differences in smoking across social groups [9]. To date, the literature on the impact of family life transitions on smoking behaviors is very limited. The present study is therefore an attempt to fill this gap by focusing on the impact of family transitions on smoking behavior, especially in relation to couple formation, first childbirth, and separation.

Empirical findings on smoking behaviors and family events are mostly based on contemporaneous correlations in cross-sectional data, with of a focus on the effect of family status rather than on family events. A protective effect of being in a couple on smoking has been shown with a greater influence of living with a partner for men, whereas women are more affected by separation [10,11]. Concerning the birth of a child, the literature shows few changes in smoking of men in comparison with women. Studies of men’s smoking behaviors at the time of the birth of their child are rare and show little changes in smoking behavior or only among the best-educated [12], with the exception of changes in their habits such as not smoking in the home [13].

One shortcoming of available studies is that they have considered family transitions as discrete events and only considered the subsequent changes. Viewing family transitions as discrete events per se is questionable, as those transitions are the outcome of a long process and their consequences develop over time. For instance, marital relations start to deteriorate long before the partners break up, and there is often a long time elapsing from the initiation of the project of having a child to the actual birth [11]. Family transitions may therefore lead to changes in behavior occurring before the happening of the event (‘anticipation effects’). They may also lead to changes after the transition has taken place, and those may be temporary or permanent (‘adaptation effects’). Another aspect which has hardly been addressed is that of the gendered dimension of the long-term processes. In other words, do transitions within the family similarly influence men’s and women’s health behaviors, and if not, what are the gender-specific patterns?

In this paper, we investigate the question of family life transitions and smoking with a perspective integrating both the time and gender dimensions. We are specifically interested in the roles of partnership, separation or childbirth as transitions triggering changes in terms of smoking uptake or cessation and in the gender-specific dynamics in this respect. Furthermore, we enlarge the observation window by considering both the three-year period preceding the actual transitions and the three-year period following them, in order to visualize the unfolding of the processes, from anticipation of impending changes to adaptation to different family life circumstances, be they positive (partnership consolidation, birth of a child) or negative (partnership breakdown), or both at the same time. To conduct this analysis, we use the Santé et Itinéraires Professionnels (SIP) survey conducted in 2006 in France, which provides both retrospective information on the timing of family life events and age of smoking initiation and eventual cessation.

## 2. Methods

### 2.1. Empirical Strategy

The aim of this paper is to estimate the impact of family events on smoking trajectory, and particularly the changes in smoking behavior before (anticipation), during (contemporary), and after (adaptation) the transition to a different family situation.

#### 2.1.1. A General Approach

We favor a discrete time approach with panel econometric strategies to distinguish between individual variations and within individual variations in longitudinal data structure. This strategy is usual in multilevel analysis and estimates both within and between individual effects by including individual means of time-varying variables, called within-between estimator or Mundlak specification in the econometric literature [14,15]. The formulation allows to test within and between effects in the same model. An alternative approach often used in a linear model is to estimate within effects separately using a fixed effects model. For the case of a binary outcome variables, such as smoking status, we favor the within-between strategy instead of the conditional logit fixed effects model that would have excluded individuals without longitudinal variation. This approach has the advantage of including ever-smokers and never-smokers in the analysis.

We consider a discrete-time logistic regression of smoking with the following formulation of the latent variable of smoking for the individual *i* at age *t*

(1) $${S_{it}}^{*}=\alpha \cdot X_{it}+\beta \cdot E_{it}+\delta \cdot \bar{E_{i}}+\gamma \cdot Z_{i}+w_{i}+e_{it}$$

with *i* = 1,…, N and *t* = 17,…T. Where $X_{it}$ is a vector of time-varying individual variables, $E_{it}$ is a vector of time-varying individual family variables (i.e., living with a partner, having a first child, being separated), $\bar{E_{i}}$ is a vector of individual means of the family variables, $Z_{i}$ a vector of fixed individual control variables, $w_{i}$ an individual specific effect of unobserved variables, and $e_{it}$ an idiosyncratic errors term.

In this model, the successive observations for a given individual from the retrospective age of 17 to the age at the time of the survey are not independent. Consequently, the logistic model was estimated with heteroscedasticity adjustment on individual clusters.

#### 2.1.2. A Specific Approach to Measure Leads and Lags

The general approach presented before assumes a contemporary relationship between variables which could be very restrictive. Here, we propose a specific approach to measure lead (anticipation) and lag (adaptation) effects of family transitions on smoking in addition to contemporary effects. This approach estimates the effect of life events on smoking behaviors up to three years before and after the event. It has been used in the context of the study of the effect of life events on life satisfaction [16,17,18], alcohol consumption [19] and more generally in the context of treatment effect analysis of public policy for example [20].

We propose to consider a three-year window around life events, with dummies of leads effect (three years before, two years before, one year before), the contemporary effect and dummies of lags effect (one year after, two years after, three or more years after)

(2) $${S_{it}}^{*}=\alpha \cdot X_{it}+\overset{3}{\underset{j=1}{\sum }}\beta_{t-j}\cdot E_{it-j}+\beta_{t}\cdot E_{it}+\overset{3}{\underset{j=1}{\sum }}\beta_{t+j}\cdot E_{it\mp j}+\gamma \cdot Z_{i}+\delta \cdot \bar{E_{i}}+w_{i}+e_{it}$$

We assume here a reference category for the interpretation of this dynamic, which is “being at least three years before the occurrence of the event” (including never experiencing it).

The effect of the different transitions are estimated jointly in the same model to adjust for successive events. We estimate a model for smoking initiation and another for cessation, separating men and women in each of them.

### 2.2. Data

This empirical work is based on the data derived from the national representative SIP (Santé et Itinéraire Professionnel) survey, performed by the French Ministries of Labor and Health (DARES and DREES), the French Center for Employment Studies (CEE) and the French National Institute of Statistics and Economic Studies (INSEE). This study uses public data collected in a survey for official statistics. All subjects gave their informed consent to participate in the survey; the data collection protocol and the questionnaire were approved by the Commission Nationale de l’Informatique et des Libertés (National Commission for Liberty and Informatics) (No. 1179915). This survey conducted in France in 2006–2007 is retrospective and jointly accounts for health events and the career paths of individuals. In 2006, households were randomly selected from the 1999 census, which was updated for new housings, and in each household, one individual aged 20–74 years was randomly selected for interview. Finally, 13,648 men and women from the general French population were interviewed at home by a trained interviewer. The participation rate was 76%. The scope of the analysis is restricted to individuals aged between 25 and 74 at the time of the survey, which amounts to 4989 men and 5812 women.

The questionnaire made it possible to retrospectively date the age of initiation of daily smoking and the age at the time of smoking cessation (“*Si vous fumez actuellement tous les jours, depuis quel âge?*”*/*“*Si auparavant vous fumiez tous les jours, de quel âge à quel âge*?”). Assuming the continuity of smoking from the age the respondent started until the age they quit (ex-smokers) or the year of the survey (current smokers), we constructed a smoking binary indicator for every year between the age of 10 and the age at interview, whose value is one if a respondent smoked or zero otherwise. 

We use information collected on a biographical grid to construct the family status variables. Partnership episodes are reported and allow to date the age at union and separation periods (“*Avez-vous déjà vécu en couple? Si oui, situez la ou les périodes*”). The questionnaire defined a partnership episode as a cohabitation period (not necessarily a marriage) during at least one year. Shorter episodes are reported only in the case of the birth of a child. We also used information on the age at first birth (“*Avez-vous eu ou bien adopté des enfants? Si oui, notez les années de naissance*”).

The distributions of age at smoking initiation and cessation and of family events are presented in Supplementary Materials Table S1 (see Figures S1 and S2 in Supplementary Materials for graphical representations). This retrospective information is used to construct a person-years database of smoking status, family status and other time-varying or fixed individual variables. Given the age of the first family events, we restricted the person-year database of analysis from 17 years old to 50 years old. Adjustment was made for activity status as a time-varying variable in five categories: long-term employment (five years or more), short-employment (less than five years, or inactivity and unemployment of less than one year), unemployment (more than one year), inactivity (more than one year), and schooling. The following variables were also adjusted for: social class from last or current (six categories); number of major life events during childhood (handicap or serious illness, health problems or death of a relative, family conflict or separation, violence, financial difficulties, etc.); educational level; migration status; residence in a rural municipality at the time of the survey; birth cohort; and log of cigarette prices of the period (see Table S2 in Supplementary Materials for descriptive statistics of the variables).

## 3. Results

### Anticipation and Adaptation in Smoking Behaviors with Family Transitions

First, we highlight the impact of family transitions on smoking trajectories of men and women separately (for a complete table of regression results including all covariates, see Supplementary Materials Tables S3 and S4). The results originate from different models (smoking initiation and cessation) where the effects of the family events are estimated jointly. This section presents a graphical illustration of the dynamic effect of each family transition on smoking behavior through marginal effects of the timing variables. This dynamic effect has to be interpreted in reference to the baseline level which represents at least three years before the event or never having experienced it.

**Figure 1:**
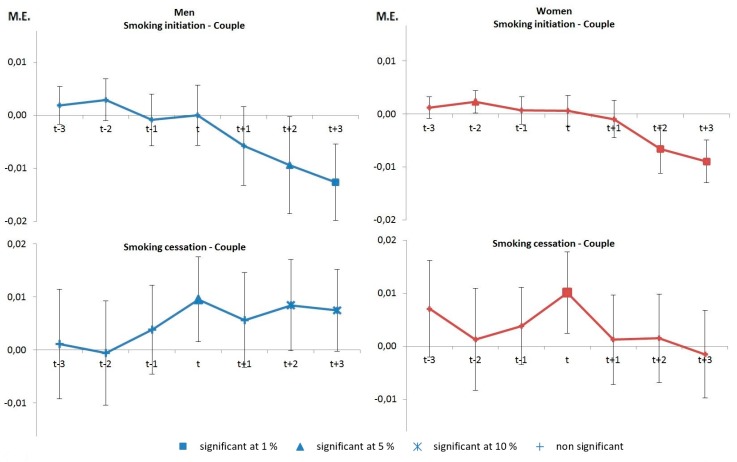
Marginal effects (M.E.) on the probability of smoking initiation and cessation around couple formation.

The graphs in Figure 1 refer to couple formation. Living with a partner has a protective effect on smoking initiation: it delays and decreases smoking initiation after three years of cohabitation for men and at least after two years for women (top panel). This protective effect of living in couple occurs as a break in behavior as smoking initiation was significantly higher for women two years before this event and as similar but non-significant effects are observed for men.

**Figure 2:**
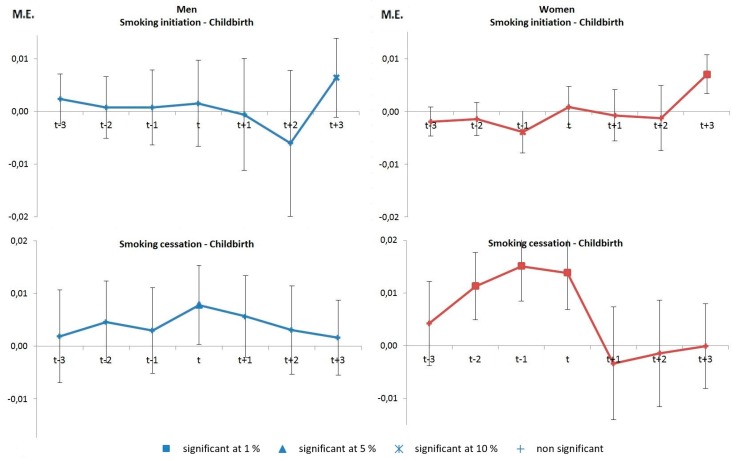
Marginal effects (M.E.) on the probability of smoking initiation and cessation around first childbirth.

Living with a partner has also an effect on smoking cessation but this effect is mainly a contemporaneous effect which is significant for men and women at the time of the event (bottom panel). There is also a long term effect of living with a partner as it increases slightly smoking cessation for men after at least two years from this transition (although no such effect is observed in women). The graphs in Figure 2 refer to first childbirth. Contrary to couple formation, the birth of a child has a different effect for men and women. Anticipation effects are visible among women two years before childbirth with a rise in smoking cessation up to two years before the birth as well a fall in initiation the year preceding the birth of a child (column 2, top and bottom panel). For men, the birth of a child has only a contemporaneous effect on smoking with a slight increase in smoking cessation at the time of birth (column 1, bottom panel). Further to that, there are adaptation effects related to the birth of a child. We find an increase in smoking initiation at least three years after first childbirth for women and to a lesser extent for men (top panel).

**Figure 3:**
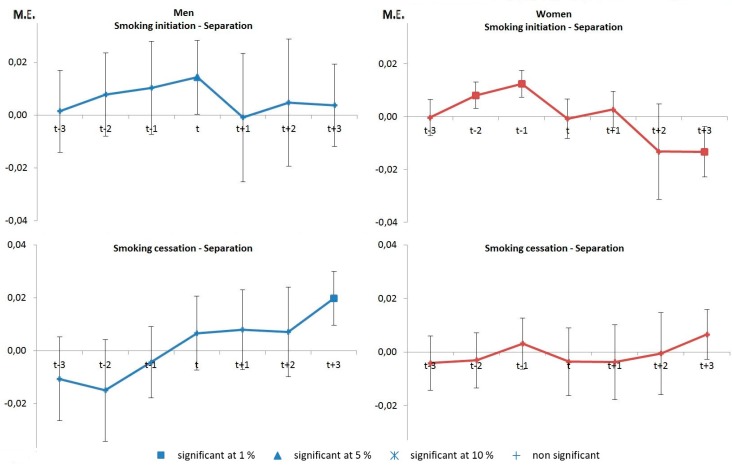
Marginal effects (M.E.) on the probability of smoking initiation and cessation around couple separation.

The dynamics of separation is also characterized by a contrast between anticipation and adaptation effects and a strong gender pattern (Figure 3). Anticipation effects precede separation for women with an increase in smoking initiation two years and one year before the separation, while the effect is only contemporaneous for men (top panel). Additionally, there is an adaptation effect, with an increase in smoking cessation after three years of separation for men (column 1, bottom panel) and a decrease in smoking initiation after three years of separation for women (column 2, top panel).

## 4. Discussion

We studied the associations between family transitions and tobacco smoking with a method that distinguishes anticipation, and contemporaneous and adaptation effects. This takes into account the fact that most of the family transitions are not precisely delimited in time nor defined with a set of criteria: there are continuums in definition and time.

### 4.1. Interpretation of the Findings

By comparing years before, during and after the family transitions, we shed light on the dynamic of the smoking behavior associated with major events of the lifecycle. Changes in smoking behavior are found over family transitions but with specific patterns of changes for men and women depending on the event.

Partnership formation was followed for both genders by a fall in smoking initiation and an immediate rise in smoking cessation. The immediate rise in smoking cessation at the time of couple formation is consistent with the hypothesis of an influence of partner. These changes may occur from the social control of health behavior or a bargaining effect between partners leading to a protective change in health behavior [21,22]. The long term effect of living with a partner is also in line with the literature that emphasizes the fact that marriage is protective against smoking [11,23] and more generally favorable in terms of health behaviors [24].

Men and women tend to behave differently around couple separation with an anticipatory increase in smoking initiation for females, while this effect is contemporaneous for men. This accords with previous studies concluding that marital disruption is connected to increased smoking risks [11,25] but we further reveal that the adaptation effects lead in the long term to a return to more balanced behaviors. We also found women more affected by separation which is consistent with the greater deleterious effect of stressful events on smoking behavior for women [26].

The first childbirth accelerates cessation and delays initiation among women, while it only has a positive effect on cessation for men, which is mainly contemporaneous and fades quickly. These results are in line with previous publications regarding anticipation of pregnancy among women and reduced effects in men [5,13]. However, this beneficial effect of childbirth may also be temporary for some women who initiate smoking or relapse after pregnancy [27,28,29]. This result can be interpreted as a response to the stress and constraints related to the joint exercise of the work and family roles [30], that would limit the protective effect of childbirth in the long term, especially for women.

### 4.2. Limitations

Our study is based on retrospective data which may lead to some biases and measurement errors. First, differential mortality among smokers could lead to an underestimation of the prevalence of smoking for people older than 70 as suggested by a study testing the validity of retrospective data on smoking [31]. This bias may be limited because only a very small part of our sample is aged more than 70. The use of retrospective data could also entail recall bias and reporting errors. It was demonstrated that these biases were lower for heavy smokers [32] but we cannot address this problem because of a lack of information on the number of cigarettes that former smokers were smoking. The reconstruction of smoking itineraries is also limited by the recall of a single smoking period per individual, not allowing for temporary quitting, which is more common among the less educated, whose attempts to quit are more numerous and less successful [33,34]. This bias could in fact minimize the short term effect of some family events or transitions as adult ex-smokers are more likely to start smoking again. Nevertheless, the use of retrospective data on smoking avoids attrition and mortality with age which can be encountered with longitudinal studies. Furthermore, the questionnaire was based on an age-event grid, a validated technique of high reliability that allows a homogenous and coherent recall of family and occupational life events [35]. In addition, the advantage of our data is that the unit of time is the year, which is shorter than what is used in the vast majority of longitudinal surveys in which subjects are surveyed every two or five years without any retrospective report of change in smoking behavior. This allows a much more detailed analysis, especially the measurement of lags and leads effects. The data were collected 10 years ago but the tobacco epidemic only marginally changed since then and it is unlikely that our estimations of relations between family events and tobacco smoking would have changed in the meanwhile.

It is worth noting that we focus on some key family events related to the life cycle, adjusting on the mean time passed in each configuration (couple, first childbirth and separation/single) during the whole life. Couple formation and separation may occur many times during life and it has been shown that multiple broken partnerships reduce the probability of smoking cessation in men [36], while it is likely that the anticipation or adaptation effect would vary over age and repetition of the same events. Yet, the succession of the events and their timing are likely to moderate the effects on tobacco smoking: for example maternal age is strongly linked to an initiation after birth [37]. Our measure of the average effects of the family events is a strong argument for the robustness of our results.

Contrarily to most papers published on the subject, we did consider cohabitation and living with a partner rather than marital status: this definition is broader and less specific, but maybe more adapted to contemporary life. Nystedt (2006) found that the cohabitation was linked to an increase in smoking compared to marriage, for both genders, which is consistent with a long term protective effect of partnership.

Those findings have relevance for both general practitioners and public policy planners, as they highlight the potential of behavioral changes in smoking at the time of life transitions. Future research is needed to study the impact of life events on different subgroups such as generations, age at family events or educational levels to better understand potential disparities and identify their behaviors when facing difficult life transitions.

## 5. Conclusions

Family life transitions influence health behaviors, leading to permanent or more temporary changes, and those changes are visible well before and sometimes well after the actual shift to a different family configuration. The anticipation effects develop during the long term process leading to those transitions, and the adaptation to this different family setting also necessitates a long period of time. This vision is a better reflection of the unfolding of health behavior changes during adult life in response to the reconfiguration and instability of close family ties. Our approach shed light on the limitation of the traditional analytical strategy that takes only the contemporary effect of the life events or family status into account and lead to miss the real timing of the changes in smoking behavior. Attending to the specific vulnerabilities related to partnership disruption and using forthcoming roles and responsibilities as a motivator for positive changes could be an important component of smoking prevention and cessation efforts.

## Supplementary Materials

The following are available online at www.mdpi.com/link/1660-4601/14/6/610/s1, Table S1: Distributions of age at smoking initiation and cessation and at family events, Table S2: Descriptive statistics, Table S3: Discrete-time logistic regression of smoking initiation—Men and Women (Odds-ratio), Table S4: Discrete-time logistic regression of smoking cessation—Men and Women (Odds-ratio); Figure S1: Prevalence of smoking over ages, Figure S2: Distribution of family events over ages.
