# Supplementary material for: Changes in Smoking Behavior over Family Transitions: Evidence for Anticipation and Adaptation Effects
Source: Int J Environ Res Public Health. 2017 Jun 7;14(6):610. doi: 10.3390/ijerph14060610 (PMC5486296; doi:10.3390/ijerph14060610)
Supplement: Supplementary file 1 [file ijerph-14-00610-s001.pdf]

Supplementary materials: Changes in Smoking Behaviour over Family Transitions: Evidence for Anticipation and Adaptation Effects

Damien Bricard, Stéphane Legleye and Myriam Khlat

Table S1. Distributions of age at smoking initiation and cessation and at family events

	Men			Women		
	**% concerned**	**Mean age**	**Median age**	**% concerned**	**Mean age**	**Median age**
Initiation	46.9	18.6	18	30.0	18.9	18
Cessation	21.5	41.3	40	10.7	36.8	35
Living with a partner	91.4	25.4	24	93.4	23.7	22
First childbirth	80.4	27.2	27	84.7	25.1	25
Separation	20.9	34.4	33	32.0	34.4	34

Table S2. Descriptive statistics

Variables	Men (N=4989)	Women (N=5812)
**Individual fixed characteristics**		
Initial smoking status at 17	22.8%	14.8%
**Years of birth**		
before 1941	13.7%	12.2%
1941-1950	22.1%	20.0%
1951-1960	24.5%	26.0%
1961-1970	23.2%	23.6%
after 1970	16.6%	18.1%
**Born abroad**	11.1%	9.8%
**Living in rural municipality**	29.1%	26.8%
**Number of life events during childhood**	2.06	2.37
**Educational level**		
Primary or no education	27.8%	27.8%
Lower or upper secondary	49.7%	44.0%
1 or 2 years higher education	9.0%	11.6%
3 or more years higher education	13.5%	16.6%
**Social class (last job)**		
Manager	4.4%	2.5%
Farmer	7.5%	4.1%
Craftmen	12.5%	9.2%
Associate professional	19.6%	20.9%
Office workers	15.2%	51.0%
Elementary jobs	40.9%	12.3%
**Individual means of time-varying variables**		
Log of cigarette prices	4.66	4.67
**Occupation**		
Long-term contract	69.0%	52.8%
Short-term contract	14.8%	14.7%
Unemployement	1.5%	2.5%
Inactivity	2.5%	16.4%
Schooling	12.2%	13.5%
**Family status**		
In a couple	61.9%	66.5%
With kids	50.6%	58.2%

Separated	3.7%	6.5%

Table S3. Discrete-time logistic regression of smoking initiation – Men and Women (Odds-ratio)

Variables	Men			Women		
	O.R	S.E.		O.R.	S.E.	
**Years of birth (ref: before 1941):**						
1941-1950	1.19	(0.14)		1.60	(0.24)	***
1951-1960	1.28	(0.18)	*	2.12	(0.35)	***
1961-1970	1.44	(0.24)	**	2.48	(0.48)	***
after 1970	1.52	(0.33)	*	2.08	(0.52)	***
**Born abroad**	0.96	(0.08)		0.81	(0.09)	**
**Living in rural municipality**	1.05	(0.07)		0.77	(0.06)	***
**Number of life events during childhood**	1.02	(0.01)		1.03	(0.01)	**
**Educational level (ref: primary or no education):**						
Lower and upper secondary	1.19	(0.09)	**	1.50	(0.15)	***
1 or 2 years hugher education	0.89	(0.13)		1.17	(0.18)	
3 or more years higher education	0.97	(0.15)		1.24	(0.21)	
**Social class from last job (ref: manager):**						
Farmer	0.75	(0.14)		0.64	(0.20)	
Craftmen	1.06	(0.15)		0.95	(0.19)	
Associate professional	1.06	(0.12)		1.04	(0.13)	
Office workers	1.01	(0.12)		0.89	(0.12)	
Elementary jobs	1.15	(0.13)		0.93	(0.15)	
**Time-varying variables :**						
age	0.62	(0.03)	***	0.71	(0.03)	***
age squared	1.01	(0.00)	***	1.00	(0.00)	***
log of cigarette prices	0.60	(0.12)	***	0.29	(0.06)	***
**Occupation (ref: long-term contract):**						
short-term contract	1.11	(0.10)		1.24	(0.14)	*
unemployement	0.81	(0.35)		0.75	(0.26)	
inactivity	1.30	(0.19)	*	1.12	(0.16)	
schooling	1.14	(0.12)		1.48	(0.18)	***
**Leads and lags Couple (ref: Couple (<t-3) or no):**						
Couple (t-3)	1.11	(0.12)		1.14	(0.14)	
Couple (t-2)	1.19	(0.14)		1.30	(0.16)	**
Couple (t-1)	0.95	(0.14)		1.08	(0.16)	
Couple (t)	1.00	(0.17)		1.07	(0.18)	
Couple (t+1)	0.71	(0.16)		0.89	(0.18)	
Couple (t+2)	0.57	(0.16)	**	0.47	(0.12)	***
Couple (t+3)	0.48	(0.10)	***	0.36	(0.08)	***
**Leads and lags First childbirth (ref: First childbirth (<t-3) or no):**						
First childbirth (t-3)	1.15	(0.16)		0.80	(0.13)	
First childbirth (t-2)	1.05	(0.18)		0.85	(0.15)	
First childbirth (t-1)	1.05	(0.22)		0.64	(0.15)	*
First childbirth (t)	1.10	(0.27)		1.10	(0.25)	
First childbirth (t+1)	0.97	(0.31)		0.92	(0.26)	
First childbirth (t+2)	0.70	(0.29)		0.86	(0.31)	
First childbirth (t+3)	1.46	(0.33)	*	2.24	(0.47)	***
**Leads and lags Separation (ref: Separation (<t-3) or no):**						
Separation (t-3)	1.09	(0.51)		0.95	(0.38)	
Separation (t-2)	1.58	(0.74)		2.49	(0.72)	***
Separation (t-1)	1.84	(0.98)		4.08	(1.19)	***
Separation (t)	2.32	(0.97)	**	0.90	(0.39)	
Separation (t+1)	0.95	(0.69)		1.35	(0.54)	
Separation (t+2)	1.32	(0.95)		0.22	(0.23)	
Separation (t+3)	1.24	(0.58)		0.22	(0.12)	***
**Individual means of time-varying variables:**						
Log cigarette prices	15.81	(19.63	**	121.53	(156.48)	***
**Occupation (ref: long-term contract):**						
short-term contract	1.89	(0.41)	***	2.68	(0.60)	***
unemployement	3.04	(1.84)	*	3.42	(1.77)	**
inactivity	1.77	(0.74)		1.21	(0.25)	

schooling	0.80	(0.42)		0.55	(0.32)	
**Family status :**						
Couple	3.26	(0.85)	***	1.94	(0.65)	**
With kids	0.65	(0.12)	**	0.76	(0.18)	
Separated	4.49	(1.52)	***	4.84	(1.81)	***
**N**	4119			5168		
**N*T**	78972			117348		
**Pseudo R^2**	0.18			0.17		

Notes: Significance levels: *** 1%, ** 5%, * 10%

Table S4. Discrete-time logistic regression of smoking cessation – Men and Women (Odds-ratio)

Variables	Men			Women		
	O.R	S.E.		O.R.	S.E.	
**Age at smoking initiation**	0.96	(0.01)	***	0.96	(0.01)	**
**Years of birth (ref: before 1941):**						
1941-1950	0.77	(0.12)	*	0.79	(0.22)	
1951-1960	0.73	(0.15)		0.57	(0.16)	**
1961-1970	1.25	(0.31)		0.95	(0.32)	
after 1970	1.92	(0.67)	*	0.97	(0.40)	
**Born abroad**	0.88	(0.12)		1.27	(0.23)	
**Living in rural municipality**	0.93	(0.08)		1.09	(0.13)	
**Number of life events during childhood**	1.01	(0.02)		1.02	(0.02)	
**Educational level (ref: primary or no education):**						
Lower and upper secondary	1.30	(0.13)	***	1.07	(0.18)	
1 or 2 years hugher education	1.29	(0.27)		1.06	(0.26)	
3 or more years higher education	1.00	(0.22)		1.98	(0.51)	***
**Social class from last job (ref: manager):**						
Farmer	0.76	(0.21)		0.65	(0.40)	
Craftmen	0.68	(0.14)	*	1.02	(0.30)	
Associate professional	0.76	(0.12)	*	1.06	(0.20)	
Office workers	0.87	(0.15)		0.88	(0.19)	
Elementary jobs	0.81	(0.13)		0.68	(0.19)	
**Time-varying variables:**						
age	1.25	(0.08)	***	1.28	(0.10)	***
age squared	1.00	(0.00)	**	1.00	(0.00)	**
Log of cigarette prices	0.91	(0.17)		2.26	(0.65)	***
**Occupation (ref: long-term contract):**						
short-term contract	0.93	(0.17)		1.00	(0.19)	
unemployement	0.83	(0.26)		1.32	(0.41)	
inactivity	0.56	(0.25)		1.04	(0.24)	
schooling	0.62	(0.23)		0.35	(0.13)	***
**Leads and lags Couple (ref: Couple (<t-3) or no):**						
Couple (t-3)	1.08	(0.42)		1.87	(0.77)	
Couple (t-2)	0.96	(0.35)		1.12	(0.49)	
Couple (t-1)	1.32	(0.42)		1.40	(0.46)	
Couple (t)	2.01	(0.61)	**	2.45	(0.84)	***
Couple (t+1)	1.51	(0.51)		1.12	(0.43)	
Couple (t+2)	1.86	(0.60)	*	1.14	(0.43)	
Couple (t+3)	1.73	(0.50)	*	0.87	(0.33)	
**Leads and lags First childbirth (ref: First childbirth (<t-3) or no):**						
First childbirth (t-3)	1.15	(0.38)		1.45	(0.52)	
First childbirth (t-2)	1.40	(0.41)		2.72	(0.78)	***
First childbirth (t-1)	1.24	(0.38)		3.81	(1.12)	***
First childbirth (t)	1.78	(0.50)	**	3.39	(1.04)	***
First childbirth (t+1)	1.51	(0.44)		0.74	(0.36)	
First childbirth (t+2)	1.25	(0.39)		0.88	(0.40)	
First childbirth (t+3)	1.13	(0.30)		0.99	(0.36)	
**Leads and lags Separation (ref: Separation (<t-3) or no):**						
Separation (t-3)	0.46	(0.27)		0.69	(0.32)	
Separation (t-2)	0.33	(0.24)		0.76	(0.35)	
Separation (t-1)	0.73	(0.37)		1.33	(0.57)	
Separation (t)	1.63	(0.86)		0.73	(0.41)	

Separation (t+1)	1.80	(1.01)		0.72	(0.45)	
Separation (t+2)	1.68	(1.07)		0.95	(0.66)	
Separation (t+3)	4.30	(1.62)	***	1.79	(0.75)	
**Individual means of time-varying variables:**						
Log cigarette prices	1.32	(2.86)		94.45	(189.12)	**
**Occupation (ref: long-term contract):**						
short-term contract	0.49	(0.18)	*	0.63	(0.30)	
unemployement	0.35	(0.38)		0.15	(0.17)	*
inactivity	1.32	(0.88)		0.48	(0.22)	
schooling	2.83	(2.30)		0.62	(0.59)	
**Family status :**						
Couple	0.87	(0.45)		1.50	(0.84)	
With kids	0.50	(0.22)		0.95	(0.53)	
Separated	0.04	(0.03)	***	0.08	(0.07)	***
**N**	2333			1734		
**N*T**	49977			33422		
**Pseudo R^2**	0.08			0.09		

Notes: Significance levels: *** 1%, ** 5%, * 10%

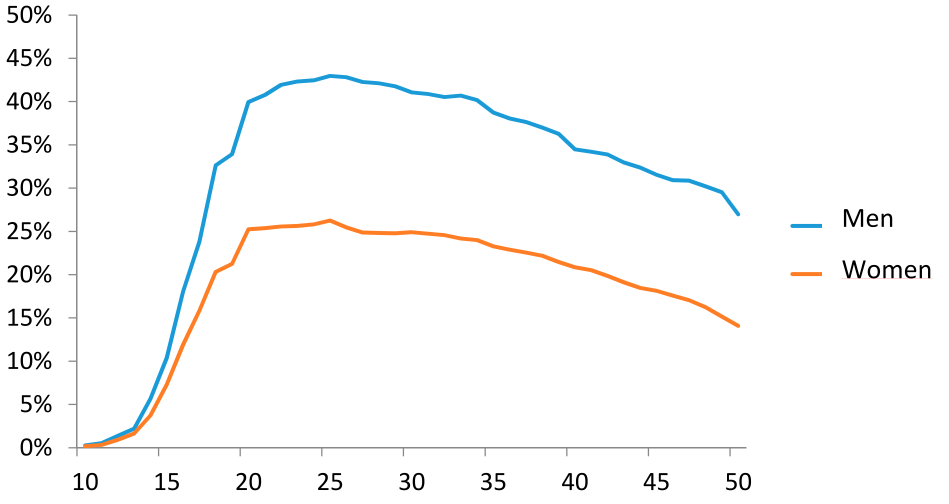

Figure S1. Prevalence of smoking over ages

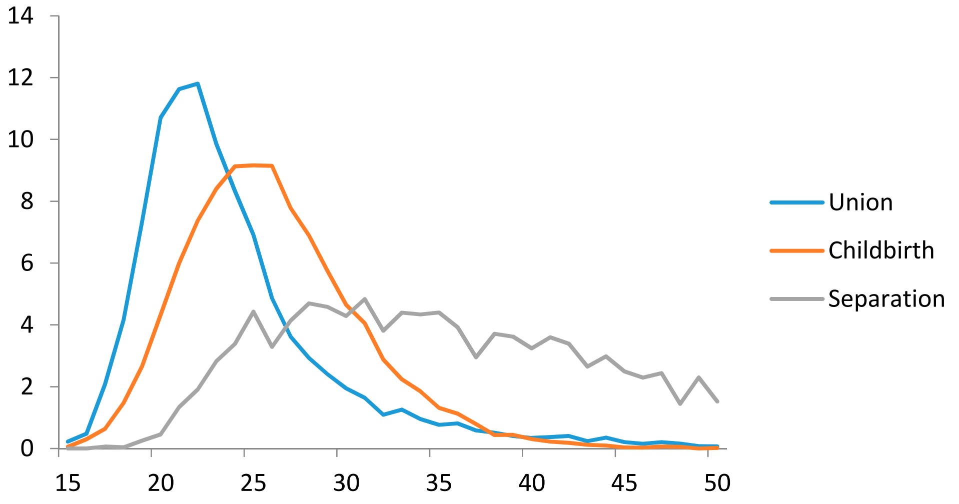

Figure S2. Distribution of family events over ages
